# Assessing frontline HIV service provider efficiency using data envelopment analysis: a case study of Philippine social hygiene clinics (SHCs)

**DOI:** 10.1186/s12913-019-4163-5

**Published:** 2019-06-24

**Authors:** Xerxes T. Seposo, Ichiro Okubo, Masahide Kondo

**Affiliations:** 1 Graduate School of Global Environmental Studies, Kyoto University, Kyoto City, Japan; 2 Environmental Health Division, Department of Environmental Engineering, Graduate School of Engineering, Kyoto University, Kyoto City, Japan; 3 Graduate School of Comprehensive Human Sciences, University of Tsukuba, Tsukuba City, Japan; 4 Yokohama City Institute of Public Health, Yokohama City, Japan; 5 Department of Health Care Policy and Health Economics, Faculty of Medicine, University of Tsukuba, Tsukuba City, Japan

**Keywords:** Data envelopment analysis, HIV, Social hygiene clinic, Philippines, Efficiency

## Abstract

**Background:**

Globally, local and frontline HIV service delivery units have been deployed to halt the HIV epidemic. However, with the limited resources, there is a need to understand how these units can deliver their optimum outputs/outcomes efficiently given the inputs. This study aims to determine the efficiency of the social hygiene clinics (SHC) in the Philippines as well as to determine the association of the meta-predictor to the efficiencies.

**Methods:**

In determining efficiency, we used the variables from two data sources namely the 2012 Philippine HIV Costing study and 2011 Integrated HIV Behavioral and Serologic Surveillance, as inputs and outputs, respectively. Various data management protocols and initial assumptions in data matching, imputation and variable selection, were used to create the final dataset with 9 SHCs. We used data envelopment analysis (DEA) to analyse the efficiency, while variations in efficiencies were analysed using Tobit regression with area-specific meta-predictors.

**Results:**

There were potentially inefficient use of limited resources among sampled SHC in both aggregate and key populations. Tobit regression results indicated that income was positively associated with efficiency, while HIV prevalence was negatively associated with the efficiency variations among the SHCs.

**Conclusions:**

We were able to determine the inefficiently performing SHCs in the Philippines. Though currently inefficient, these SHCs may adjust their inputs and outputs to become efficient in the future. While there were indications of income and HIV prevalence to be associated with the efficiency variations, the results of this case study may only be limited in generalisability, thus further studies are warranted.

**Electronic supplementary material:**

The online version of this article (10.1186/s12913-019-4163-5) contains supplementary material, which is available to authorized users.

## Background

HIV/AIDS is one of the gravest public health issues in the world. According to the 2014 Global Statistics [1], 1.2 million people died of AIDS-related illnesses, with about 36.9 million people infected with HIV, of which 2 million accounts for the annual newly-infected cases. The global response to date, through the prevention and treatment services for sufferers and population at risk, has succeeded in decreasing the number of People Living with HIV (PLHIV) steadily [2, 3].

In recent years, country-led frontline service delivery programs/interventions have been carried out to halt the epidemic across the globe [4–6]. These programs/interventions, focusing on better access to basic prevention, care and treatment (PCT) services, have catered to both the PLHIV population and key populations such as, male having sex with male (MSM), female sex workers (FSW) and people who inject drugs (PWID). Each country devised their own intervention as a response to the epidemic. In Ghana, TB integration into the HIV services through “one-stop shops” were found to have improved HIV screening [7]. While in Asia, Hong Kong and the Philippines have social hygiene clinics (SHC), which provide basic PCT services to key populations, particularly for FSW [8].

With the increasing number of HIV/AIDS-related frontline health service providers, there is a growing need to assess whether these providers have been efficiently delivering the health services subject to the available resources. In literature, effectiveness/outcomes and costing studies of the frontline HIV/AIDS services have been extensively studied independently [9–27]. However, efficiency studies, which account for both effectiveness/outcomes and costs at the same time, were relatively limited [10, 28–34]. Efficiency studies provide information about the efficient/inefficient use of finite resources, which could be subsequently utilised as potential suggestions in the improvement of both management and policy. As a tool to analyse efficiency, data envelopment analysis (DEA) has been used most often, since it is non-parametric, which imposes no restrictive hypothesis on the data generating process and requires minimal assumptions about the technology [35, 36]. Although DEA has been widely used in different disciplines [37–42], its utilisation in HIV/AIDS research is limited [28–30]. HIV/AIDS research using DEA has been instrumental to various areas across the globe as a means for resource allocation and service delivery optimisation efforts. Health centers in Rwanda were performing at an average efficiency at 78%, which suggests that there are still room for improvement in service delivery [28]. Inclusion of efficiency determination in national HIV/AIDS programmes in resources-needs estimation, using macro-level data, can narrow the spending gap in the HIV/AIDS response [29]. Lepine, et al. [35] has observed that Avahan NGOs in India could have reduced their inputs by 43% given the outputs they reached. In a more comprehensive analysis by Zanakis, et al. [43], countries with lower population density that manage to provide better health system performance and per capita support with lower GNP and better media information, tend to have lower HIV/AIDS indicators. In Kenya, Omondi Aduda, et al. [44] has observed that significant improvement in the technical efficiency among the outreach facilities were attributable to the voluntary medical male circumcision using DEA.

Most of the HIV-related DEA studies have focused on the efficiency of the delivery of health services to the general population, with little or no focus on the key populations. Key populations such as the MSM, FSW and PWID also access the same basic health services but are at higher risk of acquiring sexually transmitted diseases. Their access to these health services may vary from the general population, hence, determining the efficiency of service delivery for these populations would provide insightful suggestions to current service provider.

To the best of our knowledge, this is the first Philippine case study which aims to determine the less efficient frontline service providers, SHCs, among the aggregate and key populations, as well as to identify its possible determinants of efficiency, as a mean to improve the SHC’s operational practice compared to the efficient ones in search for better management.

## Methods

### Philippine HIV situation

As of July 2015, the Philippines has recorded a total of 27,138 HIV/AIDS cases, which has been increasing annually [45]. The country responded to the worsening HIV/AIDS epidemic, by enabling grassroots level primary health care service providers, such as the Rural Health Units (RHU) and City Health Office (CHO), to function as SHCs in providing frontline STI PCT services [46]. In the 1980s, there were roughly at total of 170 SHCs, however, the number went down to 70 SHCs due to the decrease in the accreditation of the RHU/CHO [47].

### Data envelopment analysis

In this study, DEA was used to evaluate the efficiency of the SHCs. DEA benchmarks the performance of decision-making units (DMU) such as healthcare facilities, like the SHC, taking into consideration the inputs and outputs at the same time [43, 48]. A set of efficient DMUs were identified to form the best-practice frontier, and efficiency measures were estimated relative to the remaining DMUs. After constructing an efficient frontier line, DMUs which lie on the line were considered to be efficient, while those not on the line were inefficient [49]. By benchmarking to the best performers, this will help the inefficient DMUs to improve their functional organisation based on the best performing DMUs in the frontier line [50]. DEA imposes less assumptions on the functional shape of the relationship of the outputs and the inputs [51, 52], and can handle multiple inputs and outputs at the same time. Efficiency in DEA was modeled as the maximum ratio of weighted outputs (*y*_*rk*_) to weighted inputs (*x*_*ik*_) subject to the similar ratios for every DMU be less than or equal to unity. This can be summarised in a mathematical formulation found below [36, 53–55] (Eq. 1) [56]:

1 $$ {\displaystyle \begin{array}{l}\mathit{\operatorname{Max}}\ {\theta}_k=\frac{\sum_{r=1}^S{u}_r{y}_{rk}}{\sum_{i=1}^m{v}_i{x}_{ik}}\\ {} Subject\ to\ \frac{\sum_{r=1}^S{u}_r{y}_{rk}}{\sum_{i=1}^m{v}_i{x}_{ik}}\le 1,\\ {}{u}_r\ge \varepsilon, {v}_i\ge \varepsilon \forall r,i\end{array}} $$

where,

k ≡ SHCs;

*θ* ≡ efficiency coefficient;

v_i_, i = 1, 2, …, m, are the weights assigned to the i-th inputs;

u_r_, r = 1, 2, …, m, are the weights assigned to the r-th outputs

DEA was used to facilitate the input-output modeling in determining the efficiency frontier, returns to scale and inefficient-to-efficient movement in the first stage analysis. DEA explores how output variables interacted with input variables; an expansion of the basic principle of production function taking into account the multi-output, multi-input modeling.

Due to DEA’s feature of multiple input-output analysis, it has been widely used in most operational research analyses for efficiency determination [36, 57, 58]. Recent advances in DEA have further emphasised in identifying the possible determinants of efficiency by using area-specific, meta-predictors [29, 30, 59]. The efficiency coefficients in the first stage were then regressed with the area-specific parameters using Tobit regression in determining which meta-predictors can explain the variations among the efficiencies.

### Inputs and outputs

**Fig. 1:**
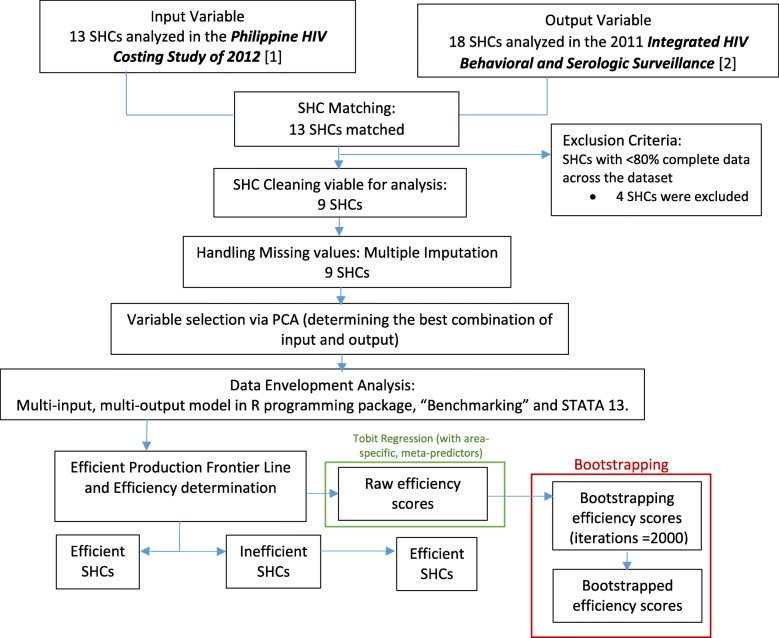
Schematic diagram of Data Processing and Analyses. Shows the flow of the robust protocols used from data matching until Tobit regression

The inputs and outputs used in this study were from secondary data sources, which are highlighted in the Additional file 1. Inputs can be in the form of unit costs or physical units [60], however, due to data availability, we were only able to acquire the latest input data of health service unit costs from the 2012 Philippine HIV Costing Study [61]. Output variables, i.e. number of people accessing a specific health service, were taken from the 2011 Integrated HIV Behavioral and Serologic Surveillance (IHBSS). The input and output variables were then managed using the robust protocol; as shown in Fig. 1. Both the 13 SHCs identified from the Philippine HIV Costing Study 2012 and 18 SHCs from the 2011 IHBSS were sampled from the remaining 70 functional SHCs across the country. After matching the input and output variables at the SHC level, we were left with 13 SHCs, having five inputs and five outputs each.

**Fig. 2:**
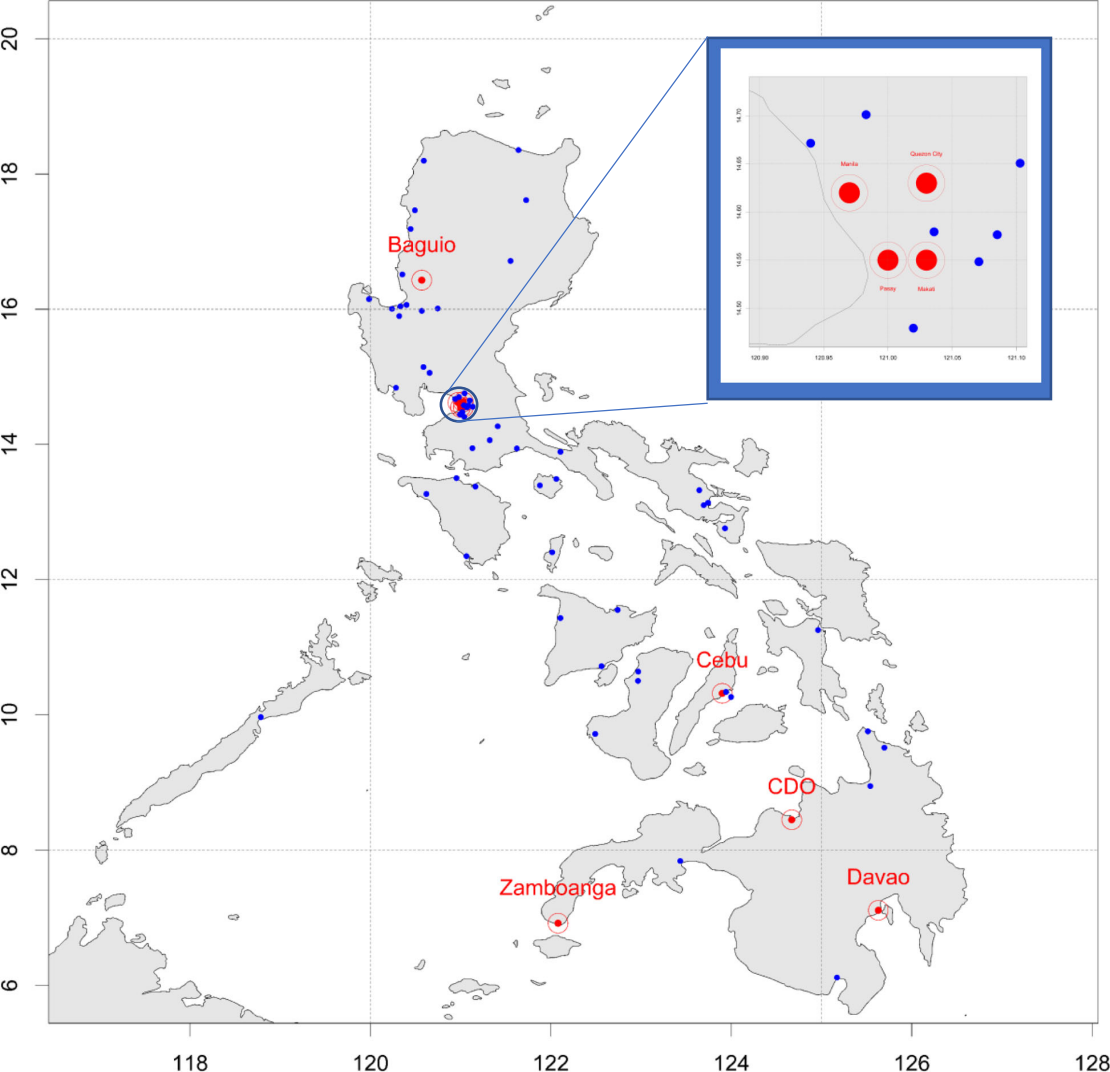
Location of the nine study SHCs. Geographical location of the operational non-study SHCs (blue dots), and the study SHCs (red dots) across the Philippines. Fig. 2 was generated using R statistical programming [86] software through the "ggmaps" package

In total there were 10 variables, with five pairs of input-output correspondence. However, DEA has been observed to have varying discriminatory power with respect to the DMUs and the input-output proportion [62]. Considerable research on the field has been done to determine the ideal proportion of DMU taking into consideration its inputs and outputs. A research by Doyle and Green [63] has noted that the number of DMUs should be at least twice that of the combined total number of inputs and outputs in order for DEA to discriminate among the efficiencies. In this study, we utilised principal component analysis (PCA) in facilitating variable dimension reduction to determine the optimum combination of inputs and outputs [64]. Based on previous studies [64, 65], PCA was noted to work well with small number of samples (*n* < 25), which is ideal for the dataset having only 9 observation data points (9 SHCs).

We used a Matlab program, PCA-DEA, by Adler and Yazhemsky [64], to perform the variable dimension reduction. In principle, the PCA replaces the original inputs and outputs with concise groups of principal components that explain the variance structure of a matrix of data through linear combinations of variables [66]. If most of the population variance can be attributed to the first few components, then those principal components can replace the original variables with minimal loss of information [67]. From the 5-input, 5-output variable in the initial dataset, the dimension reduction favoured the 3-input, 2-output combination, which was able to explain 76–80% of the variations in the component analysis on both input and output sides. Adler and Yazhemsky [64] notes that dimensionality reduction should at least be able to explain 76% [for variable returns to scale (VRS) specification] and 80% [for constant returns to scale (CRS) specification] the variation to provide good approximation of the efficiency classification.

From the 13 input-output matched SHCs, 9 SHCs were completely matched; after excluding SHCs which have less than 80% complete data. The 9 SHCs at the aggregate level were further stratified, to determine the variations between the efficiency scores if they were to be disaggregated into key populations. Although the 9 SHCs have constraints with respect to the generalisability of the results, we believe that the dataset we built based on the robust assumptions from the established secondary data sources, can provide intuitive results for well-informed policymaking for micro-level health facility managers in a resource and data-limited setting worldwide.

### Area-specific, meta-predictors

There are numerous possible candidate meta-predictors present in the secondary output data source, which may explain the variations. However, after initial diagnostics, the 55 candidate meta-predictors, which included but are not limited to education, civil status, clients, oral sex, frequency of condom use, and others, were found to have issues related to high multicollinearity, hence, we chose not to include them in the analysis. Instead, we used income and HIV prevalence, through the AMTP 5 categorisation, as meta-predictors. The AMTP 5 classification signifies the priority level of investment for a specific area, depending on how big such area contributes towards the epidemic [68]. Cities with “A” were coded with 1, while those with “B” are 0.

### SHC field location

The 9 SHCs identified in this study are located across the country with four in the national capital region. Most of the SHCs are located in highly urbanised areas of the country wherein the HIV epidemic is concentrated [69].

### Bootstrapping and Tobit regression

Even though DEA is computationally flexible, since there is no prior structural function imposed [70, 71], important issues, namely DEA technical efficiency scores being sensitive to sampling variation and that the efficiency estimates being serially correlated due to the small sample size [70, 72], can make DEA prone to biased results and overestimated efficiency scores; thereby erroneously classifying DMUs into either efficient or inefficient [70, 71, 73]. To overcome this issue, we used bootstrapping to determine whether the ranks, based on the efficiency scores, of the SHCs would vary or not.

Simar and Wilson [74] proposed a bootstrapping methodology for analysing the sampling variation and estimate the confidence intervals of the radial DEA measures ($\hat{\theta}$). Utilising homogeneity bootstrapping approach, it assumes that there is an underlying data generation process which generates the data set (x, y) from the production possibility set [64].

Bootstrapping approximates the unknown distribution using the difference between the original efficiency estimates and ‘true’ efficiency, through the distribution of the difference between the bootstrapped efficiency estimates and the original efficiency estimator, conditioned on the original data [64]. Further technical specifications of DEA-related bootstrapping are discussed in full elsewhere [64, 74, 75].

We further examined the variations with the efficiencies by using Tobit regression (in Eq. 2) to identify the possible determinants of efficiency using various area-specific meta-predictors.

2 $$ {\displaystyle \begin{array}{l}\mathrm{y}=\left\{\begin{array}{c}{y}^{\hbox{'}};0\le {y}^{\hbox{'}}\le 1\\ {}0;{y}^{\hbox{'}}<0\ \\ {}1;1<{y}^{\hbox{'}}\ \end{array}\right.\\ {}{\mathrm{y}}^{\hbox{'}}=\beta {x}_i+\varepsilon \end{array}} $$

Whereby: y is the efficiency score; *y*^′^ as the latent (unobservable) variable; *β* vector of parameters; *x*_*i*_ are the meta-predictors.

In this study, both input and output variables are secondary data sources, with no individual information, hence ethical consent is not applicable. All analyses were done using R programming and STATA 13.

## Results

Table 1 shows the summary of the inputs and outputs as well as the area-specific meta-predictors. In the aggregate population, SHCs incur the greatest cost with the HIV test 380 (± 173), which constitutes the largest percentage (84%) of input costs. While for the output side, condom access and access to gram staining services were of the same percentage (at 50%). The wide standard deviation as observed in the input side with nearly 50% of the mean, is indicative of the variations among the cost of goods/services.

**Table 1:** Summary table of the inputs, outputs and area-specific meta-predictors

SHC	^a^Input					^b^Output			
	Gram stain	Condom	HIV test	Sub-total	Sub-rank	Gram Stain	Condom	Sub-total	Sub-rank
MSM									
Baguio	20	5	250	275	8	87	75	162	3
Cagayan de Oro	16	3	122	141	5	39	55	94	7
Cebu	18	6.7	120	144.7	6	45	81	126	5
Davao	19.8	3	60	82.8	2	96	177	273	2
Makati	4	1.28	44.2	49.48	1	21	36	57	8
Pasay	27.6	13.6	96	137.2	4	42	84	126	5
Quezon City	11.6	11.5	80	103.1	3	40	113	153	4
Zamboanga	25	3	120	148	7	96	222	318	1
Sub-Total	142	47.08	892.2	1081.28		466	843	1309	
Percentage (%)	13	4	83	100		36	64	100	
Mean	17.8	5.89	112	135.2		58.3	105	163.6	
Standard deviation	7.45	4.45	63.1	66.5		29.8	63.4	88.6	
FFSW									
Baguio	20	5	250	275	8	123	45	168	6
Cagayan de Oro	16	3	122	141	5	254	152	406	2
Cebu	18	6.7	120	144.7	6	212	187	399	3
Davao	19.8	3	60	82.8	2	204	195	399	3
Makati	3.84	8.46	64.8	77.1	1	40	43	83	7
Pasay	27.6	13.6	96	137.2	4	42	21	63	8
Quezon City	11.6	11.5	80	103.1	3	132	215	347	5
Zamboanga	20.4	3	144	167.4	7	233	236	469	1
Sub-Total	137.24	54.26	936.8	1128.3		1240	1094	2334	
Percentage (%)	12	5	83	100		53	47	100	
Mean	17.2	6.78	117	141		155	137	291.8	
Standard deviation	7.02	4.10	61.2	124.3		83.7	86.8	307.2	
RFSW									
Baguio	20	5	250	275	7	300	270	570	4
Cagayan de Oro	16	3	122	141	4	298	259	557	5
Cebu	18	6.7	120	144.7	5	297	240	537	6
Davao	19.8	3	60	82.8	1	297	276	573	3
Makati	3.84	5.83	321	330.67	8	303	272	575	1
Pasay	27.6	13.6	96	137.2	3	245	266	511	8
Quezon City	11.6	11.5	80	103.1	2	261	264	525	7
Zamboanga	12.8	3	159	174.8	6	296	278	574	2
Sub-Total	129.64	51.63	1208	1389.27		2297	2125	4422	
Percentage (%)	9	4	87	100		52	48	100	
Mean	16.2	6.45	151	174		287	266	553	
Standard deviation	7.03	4.05	90.2	85.7		21.6	12.1	25.2	
AGGREGATE									
Baguio	60	15	750	825	8	510	390	900	6
Cagayan de Oro	48	9	366	423	4	591	466	1057	4
Cebu	54	20.1	360	434.1	5	554	508	1062	3
Davao	59.4	9	180	248.4	1	597	648	1245	2
Makati	11.7	15.6	430	457.3	6	364	351	715	7
Pasay	82.8	40.8	288	411.6	3	329	371	700	8
Quezon City	34.9	34.5	240	309.4	2	433	592	1025	5
Zamboanga	58.2	9	423	490.2	7	625	736	1361	1
Sub-Total	409	153	3037	3599		4003	4062	8065	
Percentage (%)	11	4	84	100		50	50	100	
Mean	51.1	19.1	380	450		500	508	1008	
Standard deviation	20.8	12.2	173	171		112	140	233	
SHC	Area-specific meta-predictors								
	^c^Income (Php)	^d^AMTP 5 Classification							
Baguio	1146	B							
Cagayan de Oro	618	B							
Cebu	6653	A							
Davao	5064	A							
Makati	10,006	A							
Pasay	2961	A							
Quezon City	11,589	A							
Zamboanga	2192	B							
Mean	5029								
Standard deviation	4094								

^a^number of people accessing the service

^b^Unit cost in Philippine Peso (Php)

^c^2012 Income (by Php 1,000,000)

^d^AMTP 5 categories are based on how big the Percentage of the epidemic a specific area contributes

Manila was excluded in the aggregate, since it has no input/output parameters for the RFSW population

Table 2 shows both raw and bias-corrected technical efficiencies derived from DEA and bootstrapping, respectively. At the aggregate level, 3 SHCs were found to be inefficient, with Pasay as the lowest with a technical efficiency score of 0.54. When disaggregated into the key populations, MSM was observed to have the least average technical efficiency score at 0.81, which seconded FFSW (at 0.84) and has still more means to improve compared to the perfect average efficiency score of RFSW (at 1.00). Though there is an overestimation of the efficiencies, with values of the bias-corrected DEA efficiencies lower than the raw technical efficiencies, the ranking of the SHCs with respect to the efficiency scores are still the same, in both the aggregate and key populations. Consistent and evident low efficiency can be observed in Pasay SHC.

**Table 2:** Raw and bootstrapped DEA efficiencies in different key populations

SHC^a^	MSM^b^			RFSW^c^			FFSW^d^			AGGREGATE^e^		
	Technical efficiency	Bias-corrected technical efficiency	95% CI	Technical efficiency	Bias-corrected technical efficiency	95% CI	Technical efficiency	Bias-corrected technical efficiency	95% CI	Technical efficiency	Bias-corrected technical efficiency	95% CI
Baguio	0.91	0.80	(0.69–0.90)	1.00	0.99	(0.99–1.00)	0.48	0.42	(0.36–0.48)	0.82	0.78	(0.73–0.81)
Cagayan de Oro	0.46	0.40	(0.33–0.45)	1.00	1.00	(0.98–1.00)	1.00	0.79	(0.65–0.98)	1.00	0.88	(0.68–1.00)
Cebu	0.50	0.44	(0.38–0.50)	1.00	0.99	(0.99–1.00)	0.93	0.80	(0.69–0.92)	0.91	0.87	(0.80–0.91)
Davao	1.00	0.78	(0.63–0.99)	1.00	0.99	(0.96–1.00)	1.00	0.78	(0.60–0.99)	1.00	0.88	(0.68–1.00)
Makati	1.00	0.78	(0.63–0.98)	1.00	0.99	(0.96–1.00)	1.00	0.77	(0.55–0.98)	1.00	0.89	(0.68–1.00)
Manila	1.00	0.78	(0.62–0.99)	–	–	–	1.00	0.77	(0.55–0.99)	–	–	–
Pasay	0.44	0.38	(0.31–0.43)	0.96	0.96	(0.95–0.96)	0.18	0.16	(0.13–0.18)	0.54	0.51	(0.45–0.54)
Quezon City	1.00	0.86	(0.75–0.99)	1.00	0.99	(0.96–1.00)	1.00	0.79	(0.64–0.99)	1.00	0.89	(0.68–1.00)
Zamboanga	1.00	0.81	(0.66–0.99)	1.00	0.99	(0.96–1.00)	1.00	0.82	(0.67–0.99)	1.00	0.91	(0.76–1.00)
Average efficiency	0.81	0.67		1.00	0.99		0.84	0.68		0.91	0.83	

^a^*SHC* Social Hygiene Clinic

^b^*MSM* Male having sex with male

^c^*RFSW* Registered Female Sex Worker

^d^*FFSW* Freelance Female Sex Worker

^e^*AGGREGATE* combined population of the MSM, RFSW and FFSW

Table 3 shows the input and output adjustments needed for the SHC to operate on the efficient frontier line. At the aggregate level, Baguio has much to improve in relation to the reduction of its input costs for gram stain (− 0.50 Php), condom (− 4.90 Php) and particularly HIV testing (− 465 Php), and likewise the increment the number of people accessing its condom services (+ 139 pax). Across the key populations, there is no clear pattern of reductions or increments among the SHCs, however, Baguio and Pasay SHCs have been observed to need more adjustments compared to the remaining SHCs. On the other hand, from among the 9 SHCs, Davao and Quezon City SHCs don’t need any change in their input or output components, which may be partially indicative, yet inconclusive signs of good management performance.

**Table 3:** Input reduction and output increment of the SHCs

SHC	MSM					RFSW					FFSW					AGGREGATE				
	I1	I2	I3	O1	O2	I1	I2	I3	O1	O2	I1	I2	I3	O1	O2	I1	I2	I3	O1	O2
Baguio	−0.18	−1.81	−172	+ 3.24e^−07^	+ 85.4	−0.20	−1.99	− 189		+ 4.78		−2.17	−77.5		+ 34.6	−0.50	−4.90	−465		+ 139
Cagayan de Oro		−0.01	−32.2		+ 10.3			−8.37		+ 18.1								− 171		+ 85.6
Cebu		−1.69	−31.3				−3.13	−27.4		+ 33.6		−2.70	−2.42e^−05^				−5.02	−153		+ 73.2
Davao																				
Makati												− 4.66	−38.3	+ 3.62	+ 28.0					
Manila						n/a	n/a	n/a	n/a	n/a				+ 22.6	+ 30.8					
Pasay	−2.65	−4.64	−7.00			−7.50	−10.2	−34.6	+ 40.5		− 1.80	−1.91			+ 9.65	−12.6	−17.2	−58.3		
Quezon City																				
Zamboanga																		− 242		

^*^I1 = Gram stain services (Php)

^*^I2 = Condom services (Php)

^*^I3 = HIV test services (Php)

^*^O1 = Number of people accessing gram stain services

^*^O2 = Number of people accessing condom services

^*^Php = currency in Philippine Peso

^*^The (−) and (+) signs, indicate the reduction and increment, respectively, in the inputs/outputs per SHC

Income and HIV prevalence, through AMTP 5 indicator, were used as the area-specific meta-predictors, instead of the output dataset’s candidate meta-predictors. HIV prevalence was observed to be negatively associated with the efficiency scores, with notable significant inverse relationship observed at the aggregate level (at − 0.60). On the other hand, income is positively associated to the efficiency scores, with a significant association observed in the FFSW (at 2.42e^− 4^). We have observed a positive association with income and negative association with HIV prevalence, as shown in Table 4. RFSW results were not shown due to the homogeneity of the efficiency scores, which can’t be analysed using Tobit regression.

**Table 4:** Tobit regression of the efficiencies with area-specific meta-predictors

Key Population	Area-specific, meta-predictor	Coefficient	*p*-value
Aggregate	Income	1.11e^−4^	0.12
	AMTP 5	−0.60	0.08^*^
MSM	Income	1.68e^−4^	0.11
	AMTP 5	− 0.72	0.17
FFSW	Income	2.42e^−4^	0.09^*^
	AMTP 5	−1.11	0.11

*MSM* Male having sex with male, *FFSW* Freelance Female Sex Worker, *Aggregate* combined population of the MSM, RFSW and FFSW, *AMTP 5* level of HIV priority based on the area-specific HIV prevalence (binary variable with low prevalence =0, high prevalence = 1)

^*^0.05 < *p*-value ≤0.10

## Discussion

The frontline service delivery is an effective response to the HIV/AIDS epidemics [4, 5]. In the Philippines, SHCs operate as one of the major frontline health service providers. In this case study, we have successfully identified the potentially inefficient use of finite resources among sampled SHCs. As exemplified by Pasay SHC, DEA was able to objectively identify its inefficiency among the SHCs, and even across the different key populations. Likewise, further analyses showed that both income and HIV prevalence were statistically related to efficiency.

Efficiency is an important index for benchmarking a DMU’s performance. Performance, according to ranking, based on either input or output variables (Table 1), would only provide limited information of the DMUs performance. Through DEA, inefficiency can be detected and be resolved by adjusting either the input or output side. At the aggregate population, in Table 2, an example of Pasay SHC, with the lowest efficiency score even after bootstrapping, can overcome this inefficiency by following the adjustments/suggestions of DEA, such as finding cheaper unit cost of inputs (e.g. 12.60 Php cheaper gram stain service and 58.30 Php cheaper HIV testing kit as shown in Table 3). Pasay SHC’s inefficiency can be attributed to the higher priced inputs in relation to the outputs (services provided) (shown in Table 3), which may be reflective of the bargaining transactions of the local government unit (LGU). The devolved functions of the government system in the Philippines is also apparent in health, particularly in the procurement of resources. LGUs have jurisdiction over the procurement of resources in their respective areas. While the procurement process is a standard protocol across LGUs, the bargaining transactions may vary, thus resulting to varied procurement prices. In achieving optimum efficiency, aside from Pasay, other inefficient SHCs in both the aggregate and key populations, such as Baguio, Cagayan de Oro, Cebu and others, can also benefit from DEA’s input reduction and output increment features (as shown in Table 3). The potential adjustments in the inputs/outputs provide an indication of future directions regarding SHC management. In Table 3, inputs can be reduced, or outputs can be increased per specific units to achieve optimum efficiency. While these DMUs are currently operating away from the efficient frontier, there are opportunities where resource use (input) and service delivery (output) can be optimised to approach the frontier, and prospectively become efficient. Specifically, the temporal changes in technology, in terms of the operational management of the respective inputs and outputs, provide an opportunity for DMUs to optimise their efficiency. In Nepal, Silwal and Ashton [76] notes the potential for hospitals to increase their outputs given the additional resources through the years. While in Greece, Polyzos [77] observed that middle-sized hospitals were able to achieve 100% technical efficiency in the span of 2009–2011 as a result of technological changes (i.e. spending cuts and constant reforms).

It should be noted that although we assume SHCs as DMUs, Philippine SHCs don’t decide independently, instead, they are under the mandate of the LGUs. In most cases, bargaining of goods/services are dependent on the LGUs, rather than the SHCs. The complicated functionality of the SHC-LGU nexus in relation to decision-making and bargaining of goods, is further affected by the uncertainty introduced by the local market. Market price dictates unit cost, which then affects the DMUs’ decision. These interrelated complexities in the functional and structural relation of SHCs, LGUs and local markets were not accounted, and brings us to one of the limitations of the study, which assumes that SHCs are DMUs, and that unit costs of goods/services from local markets have low variance in a short time horizon, thus such complexities were assumed to be null in this study.

Studies conducted in the field of HIV service facility efficiency assessment focused more on the aggregate population [28–30], which assumes a homogeneous population rather than the disaggregated, heterogeneous populations. Santos, et al. [48] notes that aggregation of data might undermine the performance of other DMUs providing the same services. In the same note, Gori, et al. [78] observed that disaggregated data provides better estimation of the effects than aggregated models. Disaggregation of service provision can provide better insights regarding the utilisation of resources per service, compared to aggregate ones [79]. This scenario was also observed in this case study, wherein among the key populations, efficiency estimates were varied, with some estimates consistently lower than the aggregate population; a case exemplified by the Pasay SHC. This suggests that Pasay SHC, should not only focus at the aggregate level, but most importantly at the disaggregated key populations in addressing the issue of inefficiency. We have also observed that SHCs performed better in providing services to the RFSW compared to the other two key populations. The difference in the performance (in terms of the efficiencies) may be related to the nature of the access by key populations. RFSW as the term implies are registered under the SHCs, whereby they are required to access the services in a timely manner. MSMs and FFSWs, on the other hand, are populations which access the services in a discretionary manner.

After bootstrapping the DEA efficiency scores (as shown in Table 2), we have determined that though there is consistent overestimation of results in the raw efficiencies, the ranking of the efficiencies even after being bias-corrected are relatively the same, which only shows that the results of this study are robust. We further examined the variations among the efficiencies by determining which exogenous area-specific, meta-predictors may be able to explain the difference among the efficiency scores. Both income and HIV prevalence (through the AMTP classification), were observed to be related with the efficiency scores. As shown in Table 4, income is positively associated with efficiency, an increase in the income of the local area may contribute to the increase in the efficiency of the SHCs. Similarly, if there is a larger proportion of income, this may translate to higher allocation for the specific programs, such that of HIV PCT [80, 81]. The devolved system of government of the country gives LGU exclusive allocative powers with respect to the management of its territorial resources as well as its own transactions. The crude assumption of greater income equates greater allocation is anchored on the premise of prioritisation. We can assume that everything has a proportional increase or decrease based on income and the level of prioritisation.

On the other hand, high prevalence areas have been significantly associated to less efficiency, in the aggregate population, as shown in Table 4. The same trend was observed in the statistically non-significant coefficients of MSM and FFSW. This inverse association is an eventual paradox to the usual understanding of the economies of scale. We believe that this paradox might have been due to two following limitations of the data sources: 1) the nature of the data, leaning towards a capital-intensive perspective, and 2) non-inclusion of treatment unit costs due to lack of complete data across SHCs. The capital-intensive approach did not include the number of health personnel in the SHCs. Highly prevalent areas may have greater number of service personnel, but since the available data included only the service delivery time and the salary wage per health personnel, labour, such as actual number of health service providers, were not accounted. Likewise, after thorough data management, most treatment variables 1in the input data source were not included in the study, as only few SHCs have sufficient unit cost data for treatment services. This may be attributable to the paradox, whereby highly prevalent areas may provide more treatment services, but the model was only able to capture the prevention and control intervention components.

With the various data management techniques used in this study, caution should be regarded with the limitations in the 1) specifications of the protocols, 2) generalisability of the results, and 3) data availability. The protocols in data generation, data matching, data imputation, and variable selection are based on the published literature and the observed plausibility for the analysis to be carried out. Since this study is a case study of the Philippine SHCs, the number of SHCs used in this study compared to the 70 SHCs all over the country may prove that the results acquired can’t be fully generalised with other units. We also acknowledge the possibility that the locations whereby the data are available may already be performing better than the rest of the SHCs with no available data, which, in turn may bias the results towards efficient performers. Though this may be the case with the current data source, there is also another possibility whereby the SHCs with no data may perform better than the study SHCs. While this possibility is plausible, we can’t ascertain the validity of this assumption especially with the limited data. As mentioned earlier, some results of this study are constrained by the data availability of actual number of health service personnel and the complete unit cost data of the SHCs. The inclusion of these variables into DEA warrants further investigation.

## Conclusions

We were able to determine the inefficiently performing SHCs in the Philippines. Though currently inefficient, these SHCs may adjust their inputs and outputs to become efficient in the future. While there were indications of income and HIV prevalence to be associated with the efficiency variations, the results of this case study may only be limited in generalisability, thus further studies are warranted.

### Operational definitions

- Meta-predictor – variables which include income and HIV prevalence, which serve as area-specific characteristics.
- Registered Female Sex Workers (RFSW) – Born female, 15 years or older, who accepted payment (cash or kind) in exchange for sex in the past 1 month and is based in an entertainment establishment registered at the local social hygiene clinic [82].
- Freelance Female Sex Workers (FFSW) – Born female, 15 years or older, who accepted payment (cash or kind) in exchange for sex in the past 1 month and is street-based or based in an entertainment establishment not registered at the local social hygiene clinic. This includes those found in all kinds of cruising sites, and those who practice indirect sex work [82].
- Males who have Sex with Males (MSM) – Born male, 15 years or older, who reported oral or anal sex with another male in the past 12 months [82].
- (Best practice) frontier – represents the maximum output that a DMU can produce from all inputs combined. DMUs on the best practice frontier are considered to be technically efficient, while those distant ones are otherwise considered to be inefficiently performing [83].
- Local Government Unit (LGU) – “are institutional units whose fiscal, legislative and executive authority extends over the smallest geographical areas distinguished for administrative and political purposes” [84].
- Constant return to scale (CRS) – assumes that the average productivity, in terms of the ratio of output (y)/input (x) is not dependent on the scale of production [85].
- Variable return to scale (VRS) – assumes a constant but also increasing or decreasing returns to scale at different scale sizes [85].

## Additional file

Additional file 1: Detailed data management and analyses protocols. (DOCX 49 kb)

## Abbreviations

DEA: Data envelopment analysis
DMU: Decision making unit
FFSW: Freelance Female Sex Workers
FSW: Female sex workers
LGU: Local government unit
MSM: Male having sex with males
PCT: Prevention, care and treatment
RFSW: Registered Female Sex Workers
SHC: Social hygiene clinic
