# Supplementary material for: Assessing frontline HIV service provider efficiency using data envelopment analysis: a case study of Philippine social hygiene clinics (SHCs)
Source: BMC Health Serv Res. 2019 Jun 24;19:415. doi: 10.1186/s12913-019-4163-5 (PMC6591825; doi:10.1186/s12913-019-4163-5)
Supplement: Supplementary file 1 — Detailed data management and analyses protocols. (DOCX 49 kb) [file 12913_2019_4163_MOESM1_ESM.docx]

***Additional file 1***

*Assessing frontline HIV service provider efficiency using data envelopment analysis: A Case Study of Philippine Social Hygiene Clinics (SHCs)*

*Input data: Philippine HIV Costing Study of 2012*

The Philippine HIV costing study of 2012 is the first study which measured the costs of services in the HIV/AIDS interventions in the country. The costing study analysed the unit cost used in the delivery of services in the key populations in the Philippines, which include: aggregated MSM and TG, RFSW, FFSW, MSW, and PWID [1].

*Output data: Integrated HIV Behavioral and Serologic Surveillance of 2011*

IHBSS is a surveillance data collected biennially to provide HIV and risk behavior prevalence data that is necessary to enable the country to monitor and respond to its epidemic [2]. IHBSS focused more on the surveillance of FSW (both RFSW and FFSW), MSM and PWID. Among the questions included in the IHBSS, service provision and access were also included, which complements the costing dataset of HIV/AIDS services with the number of people belonging to a specific key population varying per response to the services accessed.

*Matching Assumptions*

In order to further establish the compatibility of the two datasets, we matched the variables in the IHBSS dataset with that of the costing dataset using the actual names. We dropped some variables from the IHBSS dataset which are severely lacking; namely, Pap smear (in FFSW/RFSW) and lubricant (among MSM). We matched the data based on the following actual names:

| Data Matching for RFSW/FFSW | |
| --- | --- |
| **OUTPUT (IHBSS 2011)** | **COST (COSTING STUDY 2012)** |
| Accessed SHC STI services (past 12 months) | Syphilis Testing or Gram Stain or STI Post-test Counseling or Gonorrhea Tx or Non-Gonococcal Tx or Syphilis Tx |
| Received free condoms in the past 12 months (SHC, NGO) | Condom social marketing |
| % of MSM who had an HIV test in the past 12 months and got results | Pre-test counseling, HIV Test (screening) and Post-test counseling (+/-) |

| Data Matching for MSM | |
| --- | --- |
| **OUTPUT (IHBSS 2011)** | **COST (COSTING STUDY 2012)** |
| Accessed SHC STI services (past 12 months) | Syphilis Testing or Gram Stain or STI Post-test Counseling or Gonorrhea Treatment or Non-Gonococcal Treatment or Syphilis Treatment |
| Received free condoms in the past 12 months (SHC, NGO) | Condom social marketing |
| % of MSM who had an HIV test in the past 12 months and got results | Pre-test counseling, HIV Test (screening) and Post-test counseling (+/-) |

For the variable “*Accessed SHC STI services (past 12 months)*”, since all of the variables within the costing study can be an STI service, we used Gram Stain as the equivalent variable because of its mode of usage; Gram Stain was found to have the highest mode of usage among the STI services provided [1].

For the “*Received free condoms in the past 12 months (SHC, NGO)*”, condom social marketing was used.

In the last variable for the *“% of MSM who had an HIV test in the past 12 months and got results*”, we assumed four variables. Primarily because:

1. Pre-test counseling = A person needs to get a pre-test counseling to understand the reason why he or she is taking the test (this is one of the basic services as part of health education provided in a health center);
2. HIV Test (screening) = To get the HIV test;
3. HIV Test (-) = the person will be counseled if the test is negative (for a shorter time, compared to a positive result; and,
4. HIV Test (+) = the person will be counseled if the test is positive

For the Provider time, in costing the HIV pre-test counseling and post-test counseling (-/+) the salary per personnel we costed per minute. For this variable, we assumed the following:

1. When the variable indicates the usage of “and”, for example “personnel A and personnel B”, we assumed that the cost will be additive:
   - (Personnel A’s Salary/minute + Personnel B’s Salary/minute)*(Provider Time)
2. When the variable indicates the usage of “or” [1], for example “personnel A or personnel B”, we assumed that the cost will be borne by the personnel with the highest salary/minute:

- If personnel A is a physician while personnel B is a nurse, cost was calculated for this variable based on: (Personnel A’s Salary/minute)*(Provider Time)

1. When there is no indication of which personnel attended to the patient, we assumed that it is the doctor/physician who attended (maximum assumption).

After using these assumptions, we ended up having five variables which are eligible for analysis; namely, gram stain, condom, pre-test, HIV test and post-test services.

*Area-specific Meta-predictors data*

During the exploratory analysis, IHBSS has an accompanying data for the area-specific meta-predictors which include but are not limited to: age, sex, civil status, member of a social networking website, age at first sex, condom use, etc. However, due to high collinearity, we used the publicly available data of income and HIV prevalence, instead. Income has been used in other countries with regard to efficiency determination [3-5], while we used the Fifth AIDS Medium Term Plan (AMTP 5) categories to proxy for the HIV prevalence of the area; “A” is coded with 1, while “B” is with 0. AMTP 5 is the National HIV/AIDS roadmap of the Philippines [6], the categorisation of the areas in the Philippines, based on AMTP 5, are based on the area’s HIV prevalence.

*Variable Selection and Management*

All of the 9 SHCs have actual/imputed 5 inputs and 5 outputs based on data matching. However, one of the main issues of using DEA is the optimum number of inputs and outputs needed in order to carry out the analysis. Doyle and Green [7] proposes to resolve this issue by making the number of DMU, in this case the SHC, to be at least twice that of the combined total of the number of inputs and outputs in order for DEA to differentiate the efficiencies. Using the current number of inputs and outputs, it may prove to be difficult for DEA to differentiate the efficiencies, since we will have twice the input-output combination compared to the 9 SHCs, hence, we used principal component analysis (PCA) in reducing the number of inputs/outputs, to attain the optimum number of inputs and outputs [8].

From the 5 input, 5 output variable in the initial dataset, the number of eligible inputs and outputs were reduced to 3 and 2, respectively, which was observed to explain 76-80% of the variations in the component analysis on both input and output sides. Adler and Yazhemsky [8] notes that when reducing inputs and outputs, the resulting combination of the inputs and outputs should at least be able to explain 76% (for VRS specification) and 80% (for CRS specification) of the variation to provide good approximation of the efficiency classification, wherein in this case was feasible.

*Model Orientation and Analysis*

After dealing with the sufficiency of the number of inputs and outputs, we proceeded with DEA taking into consideration technical efficiency; though there might be other types of efficiency measures, we were not able to take them into account due to data limitations [9-12]. In general terms, efficiency can be related to cost (saving or minimizing), related to both cost and quality (related to optimisation), or equivalent to or directly related to overall value (Burgess, 2012). However, DEA defines efficiency as a relative measure of a DMU to the best performing DMU/s [13].

We further classified DEA efficiency measures based on the technical efficiency as a means to assess the SHCs. Technical efficiency “is the physical relation between resources and health outcome” [14]. In other words, it is when we get the maximum outcome from the set of input resources, or uses the minimum amount of inputs to produce an output [15]. We also set DEA to take into account the output-based perspective, wherein the SHCs have complete control over their outputs, and not the inputs [9, 10].

*Bootstrapping DEA*

Even though DEA has advantages with regard to being computationally flexible since there is no inherent assumption imposed [16, 17]. These has given rise to important issues, namely DEA technical efficiency scores being sensitive to sampling variation and that the efficiency estimates being serially correlated brought about by the small sample size [16, 18]. DEA has been prone to biased results and overestimated efficiency scores, which can erroneously classify DMUs into either efficient or inefficient [16, 17, 19]. In order to overcome this issue, we bootstrapped the first level DEA estimates and obtained unbiased estimates with valid confidence interval [16], as shown in Table 4.

After bootstrapping the raw efficiency scores, we have observed that there is consistent overestimation of efficiencies across the different populations as seen in Table 4. DEA tends to overestimate the efficiency scores especially when there are small observations [17, 19]. Zeng, et al. [4] have also observed these limitations, and thereby notes that caution and careful consideration should be taken in determining the number of DMUs, since DEA is sensitive to the number of observations included. The overestimation of the estimates in Table 4 are similar with those observed by Obure, et al. [18] and Mukherjee, et al. [20], wherein the raw efficiencies suffer from serial correlation, which introduces bias away from the estimates. It can be noted that among the bias-corrected efficiency scores in Table 4, only RFSW has a near to perfect efficiency performance across the various SHCs, which is indicative that the SHCs are performing well with respect to the service delivery for the RFSW population. The main difference between the bias-corrected efficiency observed between RFSW compared to the other risk populations would be the institutional support and mandate, which reinforce the access of services by the RFSW population. In the Philippines, SHCs require RFSWs to undergo medical exam, as mandated by the occupational and safety laws, as a proof of being fit to work [21]. Morisky and Urada [22] have observed that appointment-keeping ratios in the Philippines had higher rates of condom use with an odds ratio of 2.7.

Nevertheless, we observed that the ranks of the SHCs based on either raw or bias-corrected efficiency have been similar, which makes the results of this study robust.

*Tobit regression*

After bootstrapping the estimates, we have observed that there are variations with the efficiency measures, hence, we employed a second-stage using Tobit regression to identify the possible determinants of efficiency using various area-specific meta-predictors. Since the efficiency scores in DEA are restricted within a specific range, 0 ≤ efficiency score ≤ 1, Tobit regression is deemed to be appropriate in handling dependent variables with intervals [23]. Alternatives to Tobit regression also include ordinary least squares regression (OLS), however, previous research have shown that it yields inconsistent parameter estimates because the censored sample is not representative of the population; which does not take into account the underlying latent sample [23].

*Summarised study protocol*

Although the results of this study are of limited generalisability, especially for different cities and countries, which utilise different inputs and measure various outputs/outcomes of interest, this study, nevertheless, was able to showcase the robustness of DEA as a potential tool for efficiency assessment even in resource- and data-limited settings.

We started with two publicly available datasets, which we managed through the following robust protocol:

1. Matching the data using either direct or indirect context;
2. Exclusion of variables with less than 80% of the data;
3. Run multiple imputation for the remaining variables;
4. Determine the proportion of inputs to the DMUs through dimensionality reduction (PCA-DEA);
5. Run DEA;
6. Run bootstrapping for validation of the first level DEA efficiency scores; and,
7. Run a Tobit regression in order to determine the association of area-specific meta-predictors to the efficiency scores as a means for external validation.

We were able to maximise the full potential of the secondary datasets, thereby allowing DEA to evaluate the SHCs’ efficiencies amidst data constraints. We should note, however, that DEA’s management-ready results are not absolute and may vary, which means that the current best performers in the efficiency frontier line may change depending on their operationalisation in the future. In brief, the efficiency frontier line is not perfect, and that the best performing DMUs may underperform, and vice versa, which can mean that there is always a room for improvement for every DMU and a chance to become a best performer given the operationalisation of the inputs and outputs.

References

1. UNAIDS-PHL. Philippine HIV costing study: Selected HIV prevention and treatment services. Manila: Joint United Nations Programme on HIV/AIDS; 2013.

2. NEC. Integrated HIV Behavioral and Serologic Surveillance. Manila, Philippines: National Epidemiology Center; 2011.

3. Zeng W, et al. Efficiency of HIV/AIDS health centers and effect of community-based health insurance and performance-based financing on HIV/AIDS service delivery in Rwanda. Am J Trop Med Hyg. 2014;90(4):740-6.

4. Zeng W, et al. Resource needs and gap analysis in achieving universal access to HIV/AIDS services: A data envelopment analysis of 45 countries. Health Policy Plan. 2015.

5. Zeng W, et al. How much can we gain from improved efficiency? An examination of performance of national HIV/AIDS programs and its determinants in low- and middle-income countries. BMC Health Serv Res. 2012;12:74.

6. PNAC. Fifth AIDS Medium Term Plan - PHAREA. 2012.

7. Doyle J, Green R. Data envelopment analysis and multiple criteria decision-making. Omega-International Journal of Management Science. 1993;21(6):713-5.

8. Adler N, Yazhemsky E. Improving discrimination in data envelopment analysis: PCA-DEA or variable reduction. Eur J Oper Res. 2010;202(1):273-84.

9. Po R-W, et al. A new clustering approach using data envelopment analysis. Eur J Oper Res. 2009;199(1):276-84.

10. Cotte Poveda A. Estimating effectiveness of the control of violence and socioeconomic development in Colombia: An application of dynamic data envelopment analysis and data panel approach. Soc Indic Res. 2012;105(3):343-66.

11. Charnes A, et al. Measuring the efficiency of decision making units. 1978.

12. Akazili J, et al. Using data envelopment analysis to measure the extent of technical efficiency of public health centres in Ghana. BMC Int Health Hum Rights. 2008;8:11.

13. Cook WD, et al. Data envelopment analysis: Prior to choosing a model. Omega. 2014;44:1-4.

14. Palmer S, Torgerson DJ. Definitions of efficiency. BMJ. 1999;318(7191):1136.

15. Coelli T, et al. Technical, allocative, cost and scale efficiencies in Bangladesh rice cultivation: A non-parametric approach. Journal of Agricultural Economics. 2002;53(3):607-26.

16. Song ML, et al. Bootstrap-DEA analysis of BRICS' energy efficiency based on small sample data. Appl Energ. 2013;112:1049-55.

17. Hawdon D. Efficiency, performance and regulation of the international gas industry - a bootstrap DEA approach. Energ Policy. 2003;31(11):1167-78.

18. Obure CD, et al. Does integration of HIV and sexual and reproductive health services improve technical efficiency in Kenya and Swaziland? An application of a two-stage semi parametric approach incorporating quality measures. Social Science & Medicine. 2016;151:147-56.

19. Halkos GE, Tzeremes NG. Industry performance evaluation with the use of financial ratios: An application of bootstrapped DEA. Expert Syst Appl. 2012;39(5):5872-80.

20. Mukherjee K, et al. Explaining the efficiency of local health departments in the US: an exploratory analysis. Health care management science. 2010;13(4):378-87.

21. Simbulan NP. Mainstreaming the rights based approach in HIV/AIDS prevention: Learning experiences from the Philippines. Learning and Empowerment: Key Issues in Strategies for HIV/AIDS Prevention2004.

22. Morisky DE, Urada LA. Organizational policy recommendations for control of STI/HIV among female sex workers in China: Regular examination of workers in social hygiene clinics. AIDS Care-Psychological and Socio-Medical Aspects of AIDS/HIV. 2011;23:83-95.

23. Cameron AC, Trivedi PK. Microeconometrics using STATA. USA: Stata Press; 2009.
